# Revolution in abortion care? Perspectives of key informants on the importance of abortion method choice in the era of telemedicine

**DOI:** 10.1080/26410397.2022.2149379

**Published:** 2023-03-06

**Authors:** Katy Footman

**Affiliations:** PhD Candidate, London School of Economics, London, UK. *Correspondence*: k.footman@lse.ac.uk

**Keywords:** abortion, patient choice, key informants, telemedicine, medical abortion

## Abstract

Patient choice of medical or surgical abortion is a standard of quality abortion care, but the choice of surgical abortion is constrained in England and Wales, particularly since the COVID-19 pandemic and introduction of telemedicine. This qualitative study explored the perspectives of abortion service providers, managers, and funders on the need to offer a choice of methods within early gestation abortion services in England and Wales. Twenty-seven key informant interviews were conducted between August and November 2021, and framework analysis methods were used. Participants presented arguments both for and against offering method choice. Most participants felt that it was important to maintain choice, although they recognised that medical abortion suits most patients, that both methods are very safe and acceptable, and that the priority for abortion services is to maintain timely access to respectful care. Their arguments related to practicalities around patient needs, the risk of reinforcing inequalities in access to patient-centred care, potential impacts on patients and providers, comparisons to other services, costs, and moral issues. Participants argued that constraining choice has a greater impact on those who are less able to advocate for themselves and there were concerns that patients may feel stigmatised or isolated when unable to choose their preferred method. In conclusion, although medical abortion suits most patients, this study highlights arguments for maintaining the option of surgical abortion in the era of telemedicine. More nuanced discussion of the potential benefits and impacts of self-management of medical abortion is needed.

## Résumé

Le choix par la patiente de l’avortement médicamenteux ou chirurgical est une norme des soins de qualité en cas d’avortement, mais le choix de l’avortement chirurgical est limité en Angleterre et au Pays de Galles, en particulier depuis la pandémie de COVID-19 et l’introduction de la télémédecine. Cette étude qualitative a examiné les perspectives des prestataires, des administrateurs et des bailleurs de fonds de services d’avortement sur la nécessité d’offrir un choix de méthodes dans le cadre des services d’avortement du début de la gestation en Angleterre et au Pays de Galles. Vingt-sept entretiens avec des informateurs clés ont été menés entre août et novembre 2021, et des méthodes d’analyse du cadre ont été utilisées. Les participants ont présenté des arguments pour et contre l’offre du choix de la méthode. La plupart d’entre eux estimaient qu’il était important de maintenir le choix, même s’ils reconnaissaient que l’avortement médicamenteux convenait à la plupart des patientes, que les deux méthodes étaient très sûres et acceptables, et que la priorité des **R**arguments touchaient les aspects pratiques autour des besoins des patientes, le risque de renforcer les inégalités dans l’accès à des soins centrés sur les patientes, les conséquences potentielles sur les patientes et les prestataires, les comparaisons avec d’autres services, les coûts et les questions morales. Les participants ont avancé que la limitation du choix avait un impact plus marqué sur les personnes qui sont le moins à même de défendre leurs intérêts et ils craignaient que les patientes se sentent stigmatisées ou isolées si elles n’étaient pas en mesure de choisir leur méthode préférée. En conclusion, même si l’avortement médicamenteux convient à la plupart des patientes, cette étude met en lumière des arguments en faveur du maintien de l’option de l’avortement chirurgical à l’époque de la télémédecine. Il est nécessaire d’avoir une discussion plus nuancée sur les avantages potentiels et les répercussions de l’autogestion de l’avortement médicamenteux.

## Resumen

La elección de aborto con medicamentos o de aborto quirúrgico por la paciente es un estándar de los servicios de aborto de calidad, pero la opción de aborto quirúrgico es restringida en Inglaterra y Gales, en particular a partir de la pandemia de COVID-19 y del lanzamiento de la telemedicina. Este estudio cualitativo exploró las perspectivas de prestadores, administradores y financiadores de servicios de aborto sobre la necesidad de ofrecer ambas opciones de métodos en los servicios de aborto proporcionados al inicio de la gestación en Inglaterra y Gales. Entre agosto y noviembre de 2021 se realizaron 27 entrevistas con informantes clave y se utilizaron métodos de análisis de marco. Los participantes presentaron argumentos a favor y en contra de ofrecer opciones de métodos. La mayoría de los participantes creían que era importante ofrecer opciones, aunque reconocieron que el aborto con medicamentos es adecuado para casi todas las pacientes, que ambos métodos son muy seguros y aceptables y que la prioridad de los servicios de aborto es mantener acceso oportuno a una atención respetuosa. Sus argumentos estaban relacionados con practicalidades relativas a las necesidades de las pacientes, el riesgo de reforzar desigualdades en el acceso a la atención centrada en la paciente, posibles impactos en las pacientes y en los prestadores de servicios, comparaciones con otros servicios, costos y asuntos morales. Los participantes argumentaron que restringir las opciones tiene mayor impacto en quienes son menos capaces de abogar por sí mismas y expresaron preocupaciones de que las pacientes se sintieran estigmatizadas o aisladas al no poder elegir su método preferido. En conclusión, aunque el aborto con medicamentos es adecuado para la mayoría de las pacientes, este estudio destaca los argumentos a favor de mantener la opción del aborto quirúrgico en la era de la telemedicina. Es necesario analizar más a fondo los posibles beneficios e impactos de la autogestión del aborto con medicamentos.

## Introduction

Over the past three decades, medical abortion has transformed the nature of abortion care.^1^ A medical abortion (MA) consists of a patient self-administering two sets of pills over a 24-28 hour period, and then expelling the pregnancy vaginally, usually at home. It has widely replaced surgical abortion methods, which involve a health care professional conducting a gynaecological procedure to remove the pregnancy. Constructions of, and discourses surrounding, abortion medications have varied since their discovery^2,3^ but MA is often framed in the language of “revolution”^4–6^ and seen as an agent of social change, countering health inequities,^7,8^ and enabling women and pregnant people to exercise control of their bodies through the process of self-management.^7,9^ MA self-management is celebrated for de-medicalising abortion, reconceptualising the patient as “provider”,^10^ and shifting control away from medical institutions.^11^ By increasing accessibility of abortion care and reducing reliance on medical professionals, MA is broadly understood to have furthered abortion rights.^1,7^ In countries where abortion is restricted in law or in practice, MA’s widespread informal use has radically changed the safety of abortion by replacing less safe methods^12^ and has improved accessibility of abortion care.^13,14^ In countries with long-established legal abortion access, evidence-based clinical developments for MA have also progressively improved access: most recently, the World Health Organization (WHO) recommendation for MA to be delivered by telemedicine has removed the requirement for any in-person clinic visits at early gestations.^15,16^

Although the narratives that surround MA highlight the potential of self-management to empower patients and de-medicalise abortion, these benefits may not be realised unless MA self-management is an individual’s preference and choice.^17^ Choice of medical or surgical abortion methods has been recognised as a standard of quality care by the WHO and the National Institute for Health and Care Excellence (NICE) in England and Wales.^18–20^ This quality standard is informed by evidence that the experience of each method is very different, that women tend to have strong preferences about abortion methods, and that service acceptability is greatest when women can choose and receive their preferred method.^21–28^ Since telemedicine was introduced in England and Wales in March 2020 due to the COVID-19 pandemic,^5^ some abortion services have constrained abortion method choice, by putting an emphasis on telemedical MA when patients are informed about their options under 10 weeks.^29^ However, pre-pandemic, patient choice of surgical abortion was already found to be restricted in England and Wales by higher waiting times, limited availability of trained staff, hospital policies and higher costs.^30,31^ MA use has steadily increased in England and Wales from 43% of abortions in 2010 to 73% in 2019, and reached 88% in 2020.^32^ With telemedicine recently confirmed as a permanent feature of abortion care in England and Wales,^33^ providers will need to determine whether and how to reintroduce choice of abortion methods into their services at early gestations. Due to high pressure on health care budgets and soaring waiting lists for life-saving care in the aftermath of COVID-19,^34^ health services may face increased pressure to constrain patient choice. This may impact abortion services in particular because service quality can be more vulnerable to financial pressures when affected groups lack a strong political voice.^35^

This study aims to explore the perspectives of abortion providers, managers, and funders on the need to offer a choice of methods within early gestation abortion services in England and Wales.

## Methods

Key informant interviews (*n* = 27) were conducted between August and November 2021 with abortion service managers, commissioners* and providers. The inclusion criteria for participation were (1) worked as a provider (any cadre) of abortion care, or in the management, organisation or commissioning of abortion services in England or Wales in the past 5 years; (2) aged 18 or over; (3) gave informed consent to be interviewed and audio recorded.

Purposive, convenience sampling was used, with the aim of recruiting participants from a range of professional backgrounds, involved in abortion care provision within both the NHS and independent sector providers (ISPs) and from a variety of geographic regions. I recruited participants in several ways. An invitation to participate in the study was circulated to members of the British Society of Abortion Care Providers (BSACP) and Doctors for Choice by an email from the administrator of each network. I also directly emailed potential participants identified through web search, through review of recent conference presentations, reports, guidelines and publications, and through my prior knowledge of key actors involved in abortion in England and Wales. Additionally, an ISP staff member shared an invitation on my behalf with abortion care commissioners. I also asked participants to share information about the study with colleagues they had trained or worked with. Recruitment continued until the interviews reached saturation and until the sample was adequately varied (in terms of geography, role and sector).

Potential participants who indicated (via email or an online study form) that they were interested in taking part in the study were contacted, according to their contact preferences, with an online informed consent form which contained the full study information. I then scheduled interviews with consenting participants, and conducted interviews via phone call, Zoom or Microsoft Teams. There was no reimbursement for taking part in the study.

Twenty-seven key informants were interviewed, and their characteristics are summarised in Table 1. Many of the participants had dual roles or had experience of working in both the independent sector and the NHS. Most participants (*n* = 22) were female, and most were white (*n* = 19). Three participants were no longer working in abortion services.

**Table 1.** Characteristics of key informants (*n* = 27)

Characteristic	*n*
Role	
Doctor	9
Nurse	6
Commissioner	5
Manager	4
Manager and service provider	2
Other	1
Sector	
NHS	11
Independent	8
NHS & Independent	3
N/A	5
Region / nation	
London	8
Wales	4
National role	4
Southwest England	3
North of England	3
East of England	3
Midlands	2

Interviews generally lasted for 50–60 minutes and were semi-structured. I used a topic guide, which I had developed for each participant type. The topic guide and interview approach were piloted with two participants and then amended. Interviews were flexible, were led by the participant’s experience and were informed by gaps and themes that emerged in previous interviews. I started interviews by asking the participant why they had been interested in participating in the study, and about their professional background. Key informants were asked whether restrictions on choice of abortion method mattered, in the context of limited health system resources, and their discussion of this topic forms the main body of data under analysis in this paper. Participants were asked broadly about method choice, rather than telemedicine abortion specifically, but their responses were shaped by the recent context of abortion care in the UK and the introduction of telemedicine. Participants were also asked to discuss how abortion services had changed over the period that they had worked in abortion care, the impacts of these changes on patients, their perception of the factors that influence patient choice, how decisions about abortion methods are reached within their service, how much choice patients have within their service, the impact of funding and commissioning, the role of providers, and the future of abortion services. Participants discussed choice of abortion methods at earlier and later gestations, but this analysis focusses on abortions at early gestations (under 10 weeks), as these services are most impacted by the recent shift to telemedical abortion.

The interviews were audio-recorded, and I transcribed the recordings. Transcripts and my interview notes were imported into Dedoose^36^ and I completed an initial inductive coding of topics and themes whilst data collection was ongoing. Framework analysis methods were then used to systematically analyse the data.^37^ I developed a coding framework, based on topics identified through the initial inductive coding, which were categorised into overarching themes. The coding framework was applied to all the transcripts, and a list of excerpts for each topic were then used to chart the data and develop topic summaries.

Throughout the interview process and analysis, I tried to consistently consider my positionality on the topic, and particularly my underlying personal belief that choice is an important feature of quality abortion care. At each stage of the research, I considered how my personal beliefs and professional experiences may influence my questions and interpretation, and I selected my interview approach and analysis method with the aim of approaching the topic from an open perspective.

The research received ethical approval from the London School of Economics and Political Science Research Ethics Committee (ref: 23691, 7th June 2021).

## Results

Most participants acknowledged arguments both for and against offering clients a choice of methods, recognising both the importance of choice and the difficulty of making method choice a reality. The arguments raised by participants related to patients’ needs, inequalities, potential impacts on patients and providers, comparisons to other similar services, costs, and moral issues. Each of these issues is discussed in detail in the following sections.

There was noticeable variation between commissioners’ and providers’ perceptions of the importance of choice of abortion method. Most participants argued that, on balance, it was important to maintain choice, and this was the dominant view among providers. However, a few participants, predominantly commissioners, were more ambivalent about whether method choice was essential.

### Patients’ needs

Many of the participants argued that patient choice of abortion method needs to be retained because of practicalities around patient needs. Participants stated that the methods are not equal in terms of risk or experience, and people have fears or concerns about both methods for different reasons. Patients’ personal circumstances influence which method will better suit them, and participants raised many factors that can determine acceptability. One participant pointed out that many of these practicalities can incite a need for either a medical or a surgical abortion, and that most arguments for either method can also be used as a counterargument:

“*All the arguments that people put forward about ‘it's [MA is] good for women’. Like, you can use your own toilet, you've got your own facilities, childcare, blah de blah. On the other hand, women say exactly the same thing. ‘I don't want to use my own toilet. Don't want to have that memory down my own toilet. I can't have my kids around. My kids'll know what's happening’ … So whilst it* [MA] *suits a lot of women. It doesn't suit other women.*” [Former NHS nurse]

Practicalities noted by participants included whether patients have a safe space to expel the pregnancy, whether they have privacy and comfort at home, whether they are keeping their abortion secret, whether they want someone with them, whether they prefer to be at home, the amount of time they have available for the process and their ability to wait for an appointment. Participants also identified factors relating to the experience such as fear about side effects or about the process, anxiety levels, desire for certainty of completion, preferred level of engagement in and consciousness during the process, desire for control, desire to have/not have an in-person visit, and perceptions of invasiveness and naturalness. Other factors raised by participants included childcare responsibilities, working hours and flexibility, domestic abuse, previous personal and family/friends’ experiences of abortion and distance from a clinic.

However, whilst most participants felt choice was important, they also highlighted that MA suits, and is the preferred option for, most patients. Some participants also felt that from the perspective of patients, the main priority was speed of access, and that the method of abortion was less important:

“*To be honest for most patients really what they’re interested in is get out of the distress they’re in as quickly as possible. And ultimately whatever is the quickest thing they will opt for. So, they don’t really mind the technique.*” [ISP service manager and NHS provider]

### Inequalities

Participants described choice of methods being important due to its implications for inequalities in access and quality of care. Participants explained that inequalities currently impact whether patients can choose their abortion method, because patients are required to have prior knowledge and the capacity to advocate for their needs if they want to access surgical care:

“*People have to a) kind of know, know a bit about what they want before they commence the interaction or um b) empowered enough to say when they don't want* [MA]*. And so, yeah, you could absolutely see how certain groups of people might not be able to do that … people with language barriers … marginalised communities, very young, vulnerable in other ways*.” [ISP manager]

This was reported to limit surgical abortion access, both prior to and since the pandemic, for those who do not feel entitled to advocate for themselves or who are less able to do their own research:

“*Unless you've got a woman who is clear about her options and knows what she wants and has had the information previously and can fight her corner, then generally speaking, what was recommended* [MA] *was what they took*.” [Former ISP nurse]

“*Some people, especially those who are a bit younger, educated, like can navigate the Internet, will have done their own reading. So much more er … proactive, not like self-management, but proactive in self informing*.” [NHS doctor]

Patients are also often required to travel further for surgical care, which is less affordable for people with lower incomes, for example:

“*So if you were to say to a patient, you've got this medical treatment here that I can give you or you can go and have your general anaesthetic, and that will be an hour and a half on the train. And I don't know, £30. And then you'll have to sort out your childcare for the day or whatever. A few women would then change their mind. Do you know what, I'll have the medical because I really cannot, cannot do that, you know, afford it in time or money*.” [NHS nurse]

Several participants said they don’t perceive method preferences being linked to age or other socio-demographic characteristics in their clinical practice. However, participants did identify characteristics, linked to inequalities, that can impact method preference or acceptability e.g. not having a private or comfortable space at home, having a language barrier, being disabled or being digitally excluded.

“*People's reasons for choosing between the methods is incredibly varied, and I'm not sure that it necessarily sort of fits into particular groups … having said that, I think, you know, one could imagine that, that having a, having a medical abortion is a very different experience when you're in a, in a home where (laughs) you're not sharing your bathroom with half a dozen other people … things like the quality of your housing must have a big impact on the experience of how it feels to have a medical abortion at home, for example. So I'm sure that there's probably a correlation with socioeconomic deprivation*.” [NHS doctor]

One participant also described their experience of providing abortion to transgender patients, some of whom had preferred inpatient care “*because of the fear of what might happen to them if anything goes wrong … And they have to call an ambulance and they look like a man … ”* [Former NHS nurse].

Another participant argued that the impact of constrained choice is greatest for people who, for example, may have less social support, fewer resources or have had negative experiences from medical interactions.

“*It's always you know the most vulnerable women and … women with most needs who fall foul of these choices, because if you're well-supported and psychologically not too vulnerable … you'll put up with it won't you. You'll get round it somehow … If you don't have any support and you've got to travel or you've got to have a painful treatment at home on your own and you don't have any support … you're not very good at interacting with medical services when things go wrong, you don't always get good response from them, then it affects you much worse than it affects people who are also at the mercy of that choice but have more resource themselves.*” [NHS doctor]

There was limited counterargument to these concerns raised by participants about inequalities, but one participant discussed that most patients do have sufficient support, so MA will suit the majority:

“*But the majority of our clients are young, fit, healthy women … and a lot, most of them do have support. It's very unusual now for somebody not to be talking to their partner, boyfriend, husband, um friend, sister, mother, auntie, you know, somebody's usually spoken to somebody about their decision and they're prepared to be with them while they pass the pregnancy at home*.” [ISP manager]

However, whilst lack of access to surgical options was felt to create problems for a small minority of people, some participants thought that limited access to surgical options would most affect those who are made vulnerable by, for example, social inequalities or health disparities:

“*It is just unfortunate that rapid change has taken place and we haven't managed to embed the pathways for the minority. The majority are pretty well, you know pretty well catered for. But if you've got something wrong with you, life's quite difficult*.” [NHS doctor]

### Impact on patients and providers

Some participants argued that choice of methods was important because of the potential long-term impacts on patients. For example, patients may feel traumatised, isolated, or stigmatised when unable to choose their preferred method.

“*Without choice I think that the actual experience can be, um, distressing and, you know, who knows really what the sort of long-term consequence is on someone's, you know, mental health … I meet people fairly frequently who, who talk about, you know, how their experience of a medical abortion was something that traumatised them. You know, and I suppose I meet them frequently because our service is where they get referred to … but you know … this is not a particularly unusual experience, for a medical abortion to be unpleasant, if not traumatising*.” [NHS doctor]

A few participants cited research on the topic that has shown that patient satisfaction is associated with getting their preferred method, and that patients can feel higher anxiety or stress if they are denied a choice. Choice was argued by some participants to be particularly important for abortion care because of stigma, and the potential for MA alone at home (with or without an in-person appointment) to feel isolating if the patient doesn’t have support.

“*I think it is quite important for recovery and, um if you like integrating the experience, I think it's quite important for them to feel that they are all going down the route that they would choose, that they're not having something forced on them … if they don't want [MA], I think it's quite easy for the stigmatising aspect of abortion to be accentuated um, and their sense of guilt and isolation could be amplified*.” [NHS doctor]

One participant also highlighted the long-term impact that lack of choice can have for patients from a life course perspective:

“*When you can't access the abortion care that feels right to you – there's a cost to that. There's an individual cost to that. Do women remember difficult abortion experiences? Yes, they do … They don't forget it when they have their children, they don't forget it when their children die, they don't forget it when they go through the menopause. You know, when we work in obs and gynae, we see women the length of their reproductive life. Do women forget these things? No, they don't forget them*.”

“A couple of participants also said that it is uncomfortable and frustrating for providers when they are not able to offer patients a true choice of abortion methods, or when they “*withhold*” information about one option due to an organisational policy to offer MA as the “*default*”.

However, a couple of participants also pointed out that patients tend to have limited prior knowledge about abortion methods, and that not all patients want to be given a choice about method as some prefer to take a clinician’s advice:

“*What I actually have heard from people who have very recently accessed abortion care is some might, some might want to be told all their options, and that will help them you know with their decision making. But some, a lot of people just want to be told what to do and get on with it*.” [Commissioner]

Participants also emphasised that both methods are very safe, acceptable and effective and that some patients are grateful to be able to access an abortion at all:

“*When you don't give women a choice, they're actually just as happy, um, with the method that they've been given because they perceive it as being their choice to have an abortion rather than a choice of method as well*.” [Former NHS nurse]

There was also a misperception among some commissioners that medical abortion was a safer option, which informed their position on method choice. For example, one commissioner stated:

“*So I mean, I'm not sure I'm er totally 100 percent adamant that women must be offered a choice of medical or surgical because surgical is more of an intervention and it carries more risks medically than a medical abortion*.”

Another commissioner described how *“we all pushed for more earlier medical because well it's safer, cheaper, better for the women”.* This misperception among commissioners about medical abortion being safer was also described by a provider participant, from their previous interactions with commissioners:

“*And, and very often it it's portrayed, medical is being portrayed as being safer. You know, there's lots and lots of times I can remember over the years, of having to correct commissioners or policy documents that either state or at least imply that medical abortion is safer*.” [NHS doctor]

### Costs of services

The higher costs of delivering surgical abortion than medical abortion were raised by several participants as a key factor that has influenced the growing use of MA and declining access to surgical abortion, particularly among ISP providers and managers:

”*Medical abortion in particular, because it's provided by nurses … is known to be a less expensive service to deliver, um, to not require as much infrastructure*.” [ISP manager]

The costs of delivering surgical and medical methods across different providers are not publicly available, but in the 2019–20 national tariffs used to reimburse NHS providers for abortions under 14 weeks gestation, the tariff for a surgical abortion was £783 while the tariff for a medical abortion was £394.^38^

Several participants, predominantly NHS providers, questioned the rationale of limiting choice of abortion methods based on cost. These participants argued that the real cost difference between the methods is small within the NHS, but that the true costs to the health system are not well understood, when the costs of treating incomplete MA are considered.

“*It's, it's a, it's an individual decision and we need to have them both, basically. And whether or not there's a cost difference, if there is one, it'll be very small. And to actually balance that out with the, you know, perhaps reduced complications after, as a follow up after um MVA* [Manual Vacuum Aspiration – surgical abortion]*, it's a, it's a research project that I'm not aware has been done yet*.” [NHS doctor]

This variation between the perspectives of NHS and ISP staff was explained by the ability of NHS services to redeploy staff between services, whereas ISPs specialise in abortion and are more impacted by staffing costs. A couple of NHS participants also argued that surgical abortion skills and capacity are essential for other services such as obstetrics and miscarriage management, which should also be considered when discussing the costs of maintaining surgical services.

One of the ISP managers pointed out that, as only a small proportion of patients would choose surgical, the cost implication of maintaining choice is limited. However, other participants, including ISP managers and a commissioner, argued that the fewer surgical abortions offered, the more expensive they become to deliver, and the less sustainable the service becomes when offered in the specialist independent sector. Another participant highlighted that, although it wouldn’t necessarily be very costly to offer more surgical abortion, this issue may not be considered high priority when compared to other waiting lists:

“*I don't think it would take a lot of money to give them real choice, but I think the fragmentation of the service is so entrenched. Turning that around, yes, is not going to be a priority compared to getting through the NHS waiting lists for cancer treatment. And, and, and maybe it shouldn't be*.” [NHS doctor]

### Comparisons to other services

Several participants invoked comparisons to other services when considering arguments for or against maintaining choice, though these comparisons drew conflicting conclusions. Participants argued that in other sexual and reproductive health services there is acceptance that choice is important, for example childbirth and contraception:

“*If we put that back to obstetrics … if you have choices in the journey you made, you're far less likely to end up traumatised by your birth experience. And I think that people respect that around childbirth … It has a political agenda. It has a professional nod*.” [NHS doctor]

However, one of the ISP participants who had previously worked in obstetrics pointed out that the choice of an elective caesarean section is rarely openly offered in practice in the NHS, either.

Some participants similarly argued that in other health services (e.g. musculoskeletal services or heart surgery) you do not have a choice about your treatment and pointed out that few areas of health care have as many treatment options as abortion. However, some participants felt that choice was more important for abortion services because it is stigmatised, or because sexual and reproductive health more broadly is not curative and is more focussed on facilitating options for patients:

“*Most medicine you get into the mindset of ‘there is a problem, a disease, a tumour, you have to address that’ and it’s fairly clear what that is … Whereas in our field of work, it’s very much: there’s no right or wrong answer, there’s a series of options that are open to you. My job is to empower you to make the best decision based on your circumstances, which only you can, um, you can know*.” [ISP manager and NHS provider]

### Moral perspective

Finally, a few provider participants made arguments from a moral perspective. Given that patients will “*do anything”* to no longer be pregnant, removing patient choice from abortion care was seen to take advantage of this desperation:

“*Ultimately, I think women are happy just to be able to have an abortion if they, if they need one and have it fairly quickly and they will put up with a lot just, just to access that service. And that's partly why the service continues as it is, because they tolerate that*.” [NHS doctor]

However, another participant described themselves as “conflicted”, and while they argued that patients should be counselled through both options, they also noted that the real choice being offered by abortion services is whether to remain pregnant:

“*Although we want to offer a choice, the actual choice we're offering to women is: do you want to be pregnant or not? And then the pathway evolves out of that*.” [ISP manager]

## Discussion

This study has explored the perspectives of abortion providers, managers, and funders on the need to offer a choice of methods within early gestation abortion services in England and Wales. Most participants felt that it was important to maintain choice of abortion methods in order to meet patients’ individual needs. Participants argued that constraining choice has a greater impact on those who are less able to advocate for themselves and for whom method choice may be more important due to challenges relating to housing, travel, social support, and lack of flexibility around work or childcare responsibilities. Removing patient choice from abortion care was seen to take advantage of a patient’s urgent need to end a pregnancy and there were concerns that patients may feel stigmatised or isolated when unable to choose their preferred method. Cost-based arguments for restricting surgical abortion access were contested by participants who argued that the cost difference is small within the NHS, that the true costs of each method are poorly understood, and that surgical skills and capacity retention are essential for other obstetrics and gynaecology services such as miscarriage management. However, many of the same participants also made the point that MA suits most patients, that both methods are very safe and acceptable, and that the key priority for abortion services is therefore to maintain timely access to respectful care and to safeguard the choice to end a pregnancy.

These findings have important policy implications for abortion services in England and Wales. Participants described how abortion method choice has been constrained throughout the pandemic, with MA at home offered as the “default” option, though evidence suggests that surgical options were limited prior to the pandemic too.^30,31,39^ With the recent approval of telemedicine as a permanent feature of abortion services in England and Wales,^33^ abortion service providers and commissioners will need to decide whether and how to safeguard method choice, as the costs and infrastructure requirements for MA continue to fall.^40^ In this study, arguments were made for limiting choice, such as the higher costs of surgical abortion and the high acceptability of MA. Some participants, particularly commissioners, held an ambivalent position on maintaining method choice. These arguments made for limiting choice by those who hold decision-making roles within abortion service delivery and commissioning may, in part, explain why method choice has become increasingly constrained. However, the perspectives of most participants in this study suggest that choice of abortion methods is still important for abortion care to be patient-centred and de-stigmatising, and to avoid reinforcing inequalities in access to care. This is supported by previous international research, which has found that service acceptability is greatest when patients can choose and receive their preferred method^21–28^ and that method preference or acceptability are closely linked to characteristics such as age,^27,41–43^ education status,^27,42,44^ ethnicity,^45^ employment status,^30^ living conditions and availability of support.^30^ Furthermore, although the resource requirements of surgical abortion are higher than medical, this study suggests a more accurate costing of medical versus surgical methods is required to inform commissioning priorities, considering the hidden costs of treatment for complications and incomplete abortion.

The costs of surgical abortion could also be further reduced, enabling greater patient choice, by clarifying that nurses and midwives, as well as doctors, can provide vacuum aspiration for abortion. This practice is recommended by the WHO,^15^ and nurses and midwives provide surgical abortion in many countries.^46,47^ The 1967 Abortion Act has been interpreted to restrict surgical abortion provision to doctors, but in a recent, detailed reassessment of the relevant law and clinical evidence it was argued that this interpretation is flawed and that nurses and midwives could lawfully provide vacuum aspirations as part of a multidisciplinary team, which nurses already do in the UK for miscarriage management.^48^ Greater efficiencies may also be achieved by investing in NHS capacity to offer surgical abortion, as NHS providers in this study argued that the cost differentials between surgical and medical methods are more limited in their services, as they are already staffed and equipped to provide a wider variety of obstetric and gynaecological care. Continuing to limit surgical abortion access may otherwise reduce the imperative to train providers in these essential skills, with negative impacts on the sustainability of surgical abortion services and on the availability of wider obstetrics and gynaecology services that require the same skills.

The findings of this study also have implications for broader discourses surrounding abortion self-management. Narratives often focus on self-management’s potential to expand reproductive freedoms, reduce health inequities, and move away from medicalised, paternalistic models of care.^7,49^ This study contributes to a growing body of literature that suggest these narratives can over-simplify the contribution of MA self-management across different legal settings. For example, studies in Tanzania, Burkina Faso and Zambia^9,50,51^ have identified the limitations of MA self-management for reducing health inequities, when vendors such as pharmacists control informal access to the drugs, and when knowledge, social networks and negotiating power are needed to gain access. A qualitative study of women’s embodied experiences of abortion in Northern Ireland and the Republic of Ireland before recent legal reforms also highlighted that MA self-management may not feel empowering when it is the only option available.^52^ In the present study, key informants highlighted how patients may need to travel further, and to have prior knowledge and the capacity to advocate for their needs, if they want to access surgical care. In this environment, self-management of medical abortion may not feel empowering, particularly given that the bodily work of abortion, which is shifted from providers to patients, holds a stigmatised status in the healthcare hierarchy.^53^ Previous research from the US and the UK has also highlighted how providers play an important role in managing patients’ expectations of the MA process, so in some senses they still retain the overall framing and power within self-management models of care.^11,53^

Participants also highlighted that the preference for and acceptability of MA self-management will depend on patients’ personal circumstances, and that a wide range of practical, social, and emotional needs can inform patients’ preferences for either a medical or a surgical method. This highlights the need for discussion about the benefits of MA self-management to be nuanced, acknowledging that one fertility control method does not suit all women and pregnant people, and avoiding categorising women as one homogenous group, which can result in those who are the most privileged within that category being better served. For example, feminists have developed important critiques of the medicalisation of reproduction,^54–56^ and there may be a strong preference among some women and pregnant people to avoid medical institutions and take greater control of their own care. However, women and pregnant people may also choose to participate in processes of medicalisation (e.g. by choosing a surgical or provider-led option), for example, to achieve better experiences of health care, or to retain control of an out-of-control biological experience.^56^ Construction of MA as more “natural”,^57^ enabling women to keep “control”, can actually play into gendered expectations that women are “attuned” to their bodies and into outdated notions of “essentialist” womanhood, as identified in studies of late pregnancy detection^58^ and of “natural” versus “medicalised” childbirth.^59^ Qualitative studies in the UK and Australia have identified that ideas about rights, bodily autonomy and politics are rarely a part of the framings women use to normalise their abortion or to reject abortion stigma,^60–62^ while medical framings of abortion experiences can sometimes be helpful for women to conceptualise and legitimise their experience.^58^ Whilst advocacy for MA self-management has been critical in strengthening abortion rights, the need for patient-centred care that meets the needs of all individuals must be at the centre of advocacy efforts for abortion care.

### Limitations

The main limitation of this study is the likelihood of some selection bias within the recruitment of key informants. It was made clear during recruitment that the interview topic was abortion method choice, so participation may have been more likely from those who have a strong interest in this issue, which could have resulted in arguments for maintaining method choice being better represented. However, a range of recruitment methods were used to try to encourage diverse participation, and participants had a range of job functions, including those in management and commissioning of abortion services, who may be more affected by the arguments for limiting choice. Key informants may have been concerned about how their organisation or interests would be represented in the findings from this research, which may have influenced them to argue either for or against patient choice, though informants were assured their organisation and identity would be anonymised. This paper is limited to focus on method choice at early gestations (under 10 weeks) in the context of the recent introduction of telemedicine, but method choice at later gestations is also an important issue that requires further focus and research.

## Conclusion

In conclusion, although medical abortion suits most patients, this study highlights several important arguments for maintaining choice of abortion methods in the era of telemedicine. These include the practicalities of patient needs, inequalities in access to patient-centred care, the potential impacts on patients and providers, and moral concerns that result from denying patient choice. There is a clear need for abortion services in England and Wales to safeguard patient choice of abortion methods, which will require greater collaboration between providers, and transparent discussions between providers and funding bodies. This research also has implications for the global narratives surrounding MA self-management, which tend to portray MA as revolutionary and celebrate self-management for shifting control away from medical institutions. Discussions of the benefits of MA self-management require more nuance, as its potential to transform power dynamics may in fact depend on whether women and pregnant people have the means and power to make a genuine choice about their abortion method, instead of an absence of options.^2^

## Footnotes

* In England and Wales, commissioners work within clinical commissioning groups, local authorities or local health boards and are responsible for planning, purchasing and monitoring health care for the population within a specific geographic region.
